# Pandemic-driven healthcare disruptions and their disproportionate impact on patients with diabetes: evidence from Texas

**DOI:** 10.3389/fpubh.2025.1597297

**Published:** 2025-10-03

**Authors:** Meryem Saygili, Gultekin Gollu, Zehra Tekin

**Affiliations:** ^1^Department of Social Sciences, Economics Division, University of Texas at Tyler, Tyler, TX, United States; ^2^Department of Health Management and Policy, Georgetown University School of Health, Washington, DC, United States; ^3^Department of Internal Medicine-Endocrinology, University of Texas Southwestern Medical Center, Dallas, TX, United States

**Keywords:** COVID-19 pandemic, SARS-CoV-2 virus, healthcare disruptions, in-hospital mortality, length of stay, diabetic ketoacidosis, diabetes

## Abstract

**Background:**

Diabetic Ketoacidosis (DKA) is a life-threatening complication of uncontrolled diabetes. The COVID-19 pandemic may have increased DKA incidences and worsened outcomes both through the potential diabetogenic effects of SARS-CoV-2 infection (direct effect) and through pandemic-related disruptions in healthcare (indirect effects,) including delays in seeking or receiving care, reduced access to routine outpatient services, and strains on hospital capacity. The objective of this study is to examine changes in the frequency and outcomes of hospitalizations with DKA in Texas hospitals following the COVID-19 pandemic and to quantify the contributions of pandemic-related healthcare disruptions and SARS-CoV-2 infection. Comparisons to patients with Acute Myocardial Infarction (AMI) and Acute Kidney Injury (AKI) were made to assess the disproportionate impacts of healthcare system disruptions on DKA patients.

**Methods:**

This retrospective observational study uses regression analyses to evaluate the relative contributions of healthcare disruptions and viral infection on DKA frequency, in-hospital mortality, and length of stay. Event study and difference-in-differences models are employed to analyze whether DKA cases were disproportionately affected compared to patients with AMI or AKI. Quarterly inpatient discharge records from 2018 to 2021 are obtained from the Texas Department of State Health Services. Over 8 million discharge records are examined.

**Results:**

DKA hospitalizations increased by 8% post-pandemic, with half of the increase attributable to pandemic-induced healthcare disruptions (indirect effects.) The average mortality of patients with DKA increased by 44% compared to the pre-pandemic average. Non-COVID channels contributed to a 30% increase in mortality. Compared to AMI and AKI patients, DKA patients were disproportionately affected by pandemic-induced disruptions.

**Discussion:**

The COVID-19 pandemic significantly impacted DKA outcomes through the direct effects of SARS-CoV-2 infection and through various healthcare system disruptions—such as reduced access to routine diabetes care, delays in seeking treatment, and hospital resource constraints. Policies supporting uninterrupted diabetes management, such as telemedicine and medication access programs may reduce adverse outcomes in future health crises.

## 1 Introduction

Diabetes is one of the major public health problems in the United States and is predicted to be so in the future. Diabetic Ketoacidosis (DKA) is a life-threatening complication of uncontrolled diabetes and is most prevalent among people with type 1 diabetes. DKA develops when the body does not have enough insulin to allow blood sugar into cells for use as energy. Instead, the liver breaks down fat for fuel, a process that produces acids called ketones, which can build up to dangerous levels (1). Not taking enough insulin or missing doses can lead to DKA. Infections or other illnesses can also trigger DKA by causing the body to produce higher levels of certain hormones that counteract the effects of insulin (2).

The COVID-19 pandemic has influenced the number and severity of DKA incidences. Even though the precise mechanisms are still being discussed, the literature shows that SARS-CoV-2 is associated with more frequent DKA and worse outcomes (3, 4). Studies also reveal that DKA prolongs the resolution of the disease and results in significantly higher mortality and worse outcomes in COVID-19 patients (5–8). Studies among pediatric patients also report significantly higher DKA rate and severe DKA incidences during the pandemic (9–11). Some studies suggest the virus precipitates DKA (5, 12–18). In addition to these direct links, limited access to health care during the pandemic may also prevent patients with diabetes from receiving timely and adequate care, potentially leading to more frequent or severe DKA emergencies (19–23). Pandemic-related resource strains, including staff shortages, may further worsen outcomes (24, 25).

In this study, we examine changes in the incidence, mortality, and length of stay outcomes of DKA cases using Texas data. We also attempt to identify the factors driving these changes, whether stemming from the direct effects of the SARS-CoV-2 virus on diabetes or from pandemic-induced disruptions in the healthcare system, which we refer to as “indirect effects.” Several mechanisms may have contributed to changes in DKA incidence and outcomes through these indirect effect channels. First, delays in seeking care and care avoidance due to fear of infection or pandemic containment measures (26, 27), as well as reductions in routine and urgent outpatient visits (28–30), led to missed opportunities for timely diagnosis and glycemic control. Reduced insulin prescription use during the pandemic (29) further compromised disease management. Second, strain on hospital resources—driven by high local COVID-19 incidence and intensive care unit occupancy—likely constrained bed availability for non-COVID conditions, raising thresholds for admission and potentially increasing the severity of cases that were hospitalized. Third, pandemic-related behavioral and psychological stressors, including disruptions in diet and physical activity and increased mental health burdens (31), may have worsened glycemic control. Understanding the predominant mechanism influencing DKA outcomes is critical for patients with diabetes. If negative outcomes are primarily attributed to the “direct effect” of COVID-19 virus, it underscores the importance of prioritizing strategies to avoid viral exposure. If the adverse outcomes are primarily due to “indirect effects” related to the disruptions in the healthcare system, our findings offer significant practical and policy insights for managing similar future pandemics.

Additionally, we compare hospitalizations for diabetic ketoacidosis (DKA) with those for two other acute conditions—acute myocardial infarction (AMI) and acute kidney injury (AKI)—that are forms of chronic disease exacerbation. This comparison helps illustrate the disproportionate impact of care disruptions on individuals with diabetes. While all three conditions are acute emergencies requiring hospital-level care and may be influenced by the direct effects of the COVID-19 virus, DKA is particularly sensitive to interruptions in routine outpatient management. Unlike AMI and AKI, which are often triggered by acute physiological stress or underlying disease progression, DKA frequently arises from missed insulin doses, delays in therapy adjustments, or running out of medications—issues closely tied to consistent access to outpatient care. Although AMI and AKI may also be affected by gaps in chronic disease management, they are less directly linked to short-term disruptions in care. This distinction is especially relevant for individuals with type 1 diabetes, who require continuous and precise self-management to avoid acute metabolic decompensation.

This study is significant for several reasons. First, it attempts to distinguish the direct impact of the COVID-19 virus from pandemic-related disruptions. Second, it compares DKA patients to individuals with other acute conditions, clearly showing that DKA patients have been disproportionately affected. Third, Texas has consistently higher diabetes rates than the national average, with a widening gap over the past two decades. Additionally, Texas has the highest rate of uninsured individuals (32), making it a compelling case for study as the lack of health insurance is associated with increased risk and severity of DKA (33, 34).

## 2 Methods

### 2.1 Data and variables

We obtained quarterly inpatient hospital discharge records for Texas hospitals from the Texas Department of State Health Services (DSHS) Hospital Discharge Database (35). Our sample spans 12 quarters from the second quarter of 2018 to the first quarter of 2021. The COVID-19 pandemic spread in Texas starting in the first quarter of 2020. We focus on the pandemic period before the vaccine became widely available, and the vaccination rates were still low. The data include over 8 million discharges with an average of over 700 thousand per quarter. The data is at the hospital discharge level. This study is exempt from IRB approval because data are anonymized and publicly available.

Hospitals with zero DKA discharges in all periods (including specialty or some rural hospitals) are excluded from the sample, resulting in coverage of approximately 70–80% of hospitals.

The DKA incidence rate is calculated as the number of DKA hospitalizations per 100,000 people. Population data for Texas is obtained from the U.S. Census Bureau’s QuickFacts.

Outcome variables are DKA discharges, in-hospital mortality, and the length of stay (LOS). The unit of analysis is at the level of individual hospital discharges. The International Classification of Diseases (ICD-10) codes are used to identify diseases. Inpatient episodes with Diabetic Ketoacidosis (DKA), Acute Myocardial Infarction (AMI), or Acute Kidney Injury (AKI) are identified based on the principal or the first of secondary diagnoses. The following codes are used to identify DKA cases: E10.1, E10.10, E10.11, E11.1, E11.10, E11.11, E13.1, E13.10, E13.11, E08.1, E08.10, E08.11, E09.1, E09.10, E09.11. For AMI, the ICD 10 codes are I21, I21.0, I21.01, I21.02, I21,09, I21.1, I 21.11, I21.29, I21.1, I21.21, I21.29, I21.1, I 21.3, I21.4, I21.9, I21.A, I21.A1, I21.A9, I21 B. For AKI, the ICD-10 codes are N17, N17.0, N17.1, N17.2, N17.8, N17.9. To identify discharges with COVID-19, the primary and all secondary diagnoses (up to 24) are checked for the ICD-10 code U07.1.

Patient characteristics such as age, sex assigned at birth, race, and ethnicity as well as primary source of payment are available. All, including age, are categorical variables. Discharges are assigned to the following age categories: 0–17, 18–44, 45–64, 65–74, and 75+. The original data have more and finer age brackets; however, patients with HIV and drug/alcohol use are placed into broader age categories for confidentiality concerns. We adopted these wider categories for consistency. A small number of observations with missing age are excluded from the sample (366 out of over 8 million observations or 0.004% of the data). Patients are assigned to sex categories as Male, Female, and Other. The last category includes missing and unknown/unidentified cases. We assigned discharges to three race categories: Black, White, and Other. The last race category includes American Indian, Asian or Pacific Islander, and other races as well as discharges missing race codes. In terms of ethnicity, the discharges are classified as Hispanic and non-Hispanic. Observations with missing ethnicity information are included in the non-Hispanic category. Discharges are sorted into five insurance categories based on the primary source of payment. Medicare includes patients covered under Medicare Parts A and B; Medicaid refers to those with Medicaid coverage; Uninsured comprises self-pay, charity, indigent, or unknown payment sources; Private includes all private insurance plans. Discharges that do not fall into these categories, such as those covered by other federal programs and Veterans Administration plans, are coded as Other.

Figure A1 shows the sample construction process with details of exclusions and handling of missing data.

### 2.2 Empirical analysis

We initially analyzed the change in the DKA incidence rate in Texas by examining descriptive time trends. The DKA incidence rate is defined as the number of DKA hospitalizations per 100,000 population.

We next examined the frequency of DKA-related discharges using model 1:


(1) $$y_{\mathrm{ith}}=\alpha_{0}+\alpha_{1}{\text{Post}}_{t}+\alpha_{2}X_{\mathrm{ith}}+\gamma_{t}+\lambda_{q}+\delta_{h}+\varepsilon_{\mathrm{ith}}$$

Where *i, t,* and *h* index discharges, time periods (year-quarters), and hospitals, respectively. In Equation 1, the outcome variable is the incidence of DKA hospitalizations represented by a dummy that takes the value of 1 if the admission *i* has DKA in records. The binary variable *Post* indicates the period after COVID-19, beginning in the first quarter of 2020. This analysis provides insight into how the frequency of DKA-related discharges relative to total discharges has changed following the pandemic.

We analyzed in-hospital mortality, and the length of hospital stay (LOS) for DKA discharges using model 2:


(2) $$y_{\mathrm{ith}}=\alpha_{0}+\alpha_{1}{\text{Post}}_{t}+\alpha_{2}\text{covi}d_{\mathrm{ith}}+\alpha_{3}X_{\mathrm{ith}}+\gamma_{t}+\lambda_{q}+\delta_{h}+\varepsilon_{\mathrm{ith}}$$

Equation 2 is estimated using discharges with DKA. Similar to model 1, *i*, *t*, and *h* index discharges, time periods, and hospitals, respectively. The mortality outcome is a binary variable, indicating if in-hospital deaths occurred, and LOS refers to the number of days spent in the hospital. *Post* is a binary variable that indicates the period after COVID-19. *COVID* is another binary variable, assigned a value of one if the ICD-10 code for COVID-19 is present in any diagnosis category. The coefficient of interest is the coefficient of the *Post* variable, $\alpha_{1}$ . If the *COVID* variable accurately captures the effect of COVID-19 comorbidity on outcomes, the coefficient of the *Post* variable can be interpreted as capturing the pandemic’s indirect effect on outcomes through healthcare-related disruptions. COVID-19 cases may have been underreported, especially at the beginning of the pandemic, due to undercoding or testing issues. Thus, we also analyze excluding the first two quarters of 2020 as a robustness check. A time trend is included ( $\gamma_{t}$ ) to control for potential long-term linear trends in the outcome variables. Quarter dummies ( $\lambda_{q}$ ) are included to capture potential seasonality. We also include hospital fixed effects ( $\delta_{h}$ ) and patient characteristics (*X*) of age, sex, race, and ethnicity.

In addition to our main analysis, we compare DKA cases before and after the pandemic by excluding DKA discharges with COVID-19 comorbidity. This allows us to isolate the effects of pandemic-related disruptions in healthcare from the direct effects of the virus. While this method is subject to potential selection bias, it provides complementary evidence for the lower-bound estimate of the impact of pandemic-induced disruptions in the healthcare system. Specifically, because COVID-19 diagnoses did not exist in the pre-pandemic period, excluding COVID-positive patients from the post-pandemic sample removes any virus-related contributions to increased mortality or length of stay. However, this exclusion may also bias our estimates downward since COVID-positive patients represent a higher-risk subgroup even in the absence of the virus (36). Also, COVID-19 incidence and mortality rates were disproportionately higher among certain subgroups, such as African American and Hispanic populations, who, on average, face higher uninsured rates and consequently reduced access to healthcare (37). Thus, omitting them may lead to an underestimation of the average effect of indirect, system-level disruptions caused by the pandemic.

We use patients with acute myocardial infarction (AMI) and patients with acute kidney injury (AKI) as comparison groups to assess whether the pandemic has impacted patients with DKA differently. While we use causal inference models, our main goal with this analysis is not causal inference but to comparatively investigate how the outcomes have changed for different groups during the COVID period. We estimate models 3 and 4 separately to compare patients with DKA to those with AMI and AKI. Model 3 is similar to an event-study design that captures outcome differences over time. Model 4 is a fixed effect model that resembles a difference-in-differences framework where AKI and AMI patients are separately used as the comparison group. Each regression uses a subsample of patients with DKA and the relevant comparison group. Model 3 identifies overall differences while Model 4 sheds light on how the virus and pandemic-related indirect effects might have affected these acute emergencies differently. Discharges that have both DKA and AMI or DKA and AKI in the records are excluded from the analysis (0.43 and 3.3% of observations, respectively).


(3) $$\begin{array}{l} z_{\mathrm{ith}}=\varphi_{0}+\Phi_{1}\sum_{t=1,t\neq 7}^{12}{\mathrm{yq}}_{t}+\varphi_{2}\mathrm{DK}A_{\mathrm{ith}}+\\ \Phi_{3}\sum_{t=1,t\neq 7}^{12}{\mathrm{yq}}_{t}\times \mathrm{DK}A_{\mathrm{ith}}+\varphi_{4}X_{\mathrm{ith}}+\delta_{h}+\sigma_{\mathrm{ith}} \end{array}$$

(4) $$\begin{array}{l} z_{\mathrm{ith}}=\delta_{0}+\delta_{1}{\text{Post}}_{t}+\delta_{2}\mathrm{DK}A_{\mathrm{ith}}+\delta_{3}{\text{Post}}_{t}\\ \times \mathrm{DK}A_{\mathrm{ith}}+\delta_{4}\text{Covi}d_{\mathrm{ith}}+\delta_{5}\text{Covi}d_{\mathrm{ith}}\times \mathrm{DK}A_{\mathrm{ith}}\\ +\delta_{6}X_{\mathrm{ith}}+\gamma_{t}+\lambda_{q}+\delta_{h}+\sigma_{\mathrm{ith}} \end{array}$$

Where $z_{\mathrm{ith}}$ denotes in-hospital mortality or length of stay for discharge *i* in period *t* at hospital *h*. *DKA* is an indicator variable for discharges with a DKA diagnosis. In Equation 3, $\Phi_{1}$ and $\Phi_{3}$ are vectors of coefficients. The coefficients of interest, $\Phi_{3}$ , capture how outcomes for DKA discharges differed from those in the comparison groups in each period, controlling for individual characteristics ( $X_{\mathrm{ith}}$ ), hospital ( $\delta_{h})\;$ and time fixed effects ( ${\mathrm{yq}}_{t}$ ). The last quarter of 2019 (period 7) is excluded as the base period. In Equation 4, as previously defined, *Post* is the dummy variable for the post-COVID period capturing the indirect channels of the pandemic. *COVID* is assigned a value of one if COVID-19 is listed in discharge records, thereby controlling for the impact of COVID-19 as a comorbid condition. The interaction term, ( Post×DKA) identifies whether the pandemic-induced indirect mechanisms have changed DKA outcomes differentially in comparison to the control group’s outcomes. Its coefficient, $\delta_{3},$ is the coefficient of interest. The Covid×DKA interaction captures the differential effect of the virus among patients with a DKA diagnosis.

## 3 Results

Table A1 presents the summary statistics of all variables used in the analysis before and after the pandemic. The share of categorical variables and the averages of numerical variables, as well as the changes from the pre-COVID to post-COVID period, are reported. The table also indicates whether the differences are statistically significant based on t-tests. The number of hospitals and total discharges are also reported. The share of patients with COVID-19 comorbidity, the average mortality rates, and the average length of stay are provided for overall discharges, as well as separately for discharges with DKA, AMI, and AKI.

The share of patients younger than 17 and older than 75 decreased, while the share of patients between 18 and 74 increased. The share of Black and White patients, while the share of those in the “Other” race category, which includes missing cases, increased. The share of patients identified as Hispanic decreased. The percentage of females decreased while the percentages of Male and Other sex categories increased. The share of patients covered by Medicaid and Medicare decreased, while those with private insurance or uninsured increased. The overall mortality increased significantly by 1.06 percentage points from 1.78 to 2.84%, The average length of hospital stays also increased slightly, by about a third of a day.

The share of patients with COVID-19 comorbidity is significantly higher among discharges with DKA than those with AMI and AKI. Mortality rates are highest in patients with AMI, followed by those with AKI, and then DKA. Patients with DKA have a relatively shorter average length of stay compared to those with AMI and AKI. However, the largest increase in both mortality and length of stay after the pandemic is observed among patients with DKA.

### 3.1 Changes in DKA frequency and outcomes

An analysis of the descriptive incidence rate trend over time in Figure A2 demonstrates an increase in the DKA incidence rate following the pandemic. Specifically, the average incidence rate rose from 22.83 to 25.78 per 100,000 population, representing a 13% increase compared to the pre-pandemic period.

Figure A3 shows unadjusted DKA per 1,000 discharges, and mortality and length of stay of discharges with DKA over time. The figure plots the variables for all DKA discharges and non-COVID DKA discharges separately. The first panel shows that the frequency of DKA discharges increased dramatically with the start of the pandemic. Even for non-COVID DKA cases, the increase is significant, which indicates that both COVID-19 and indirect factors played a role. The most pronounced increase is in DKA mortality rates, affecting both overall and non-COVID cases. The average length of hospital stays also increased notably.

Tables 1–3 shows the regression results from models 1 and 2. Examining the frequencies without controls and time trend, DKA hospitalizations overall increased by 1.98 per 1,000 discharges, which corresponds to a 22% increase from the pre-pandemic levels. After adjusting for patient characteristics, hospital fixed effects, and quarter fixed effects, DKA discharges increased by 1.74 per 1,000 discharges, a 19% increase from the pre-pandemic average. When a time trend is added, the increase is approximately 8%. The coefficient estimates in columns 4–6 suggest that about half of the increase in DKA frequency is driven by non-COVID cases, which is attributable to disruptions in the healthcare system.

**Table 1:** Diabetic ketoacidosis frequency.

Variables	(1)	(2)	(3)	(4)	(5)	(6)
	DKA	DKA	DKA	Non-COVID DKA	Non-COVID DKA	Non-COVID DKA
Post	1.98*** (0.0000)	1.72*** (0.0000)	0.81*** (0.0001)	1.05*** (0.0000)	0.81*** (0.0000)	0.61*** (0.0028)
% Change	22%	19%	9%	12%	9%	7%
Observations	8,622,092	8,622,092	8,622,092	8,622,092	8,622,092	8,622,092
Controls	No	Yes	Yes	No	Yes	Yes
Time-trend	No	No	Yes	No	No	Yes
Baseline	8.95	8.95	8.95	8.95	8.95	8.95

Estimates from model 1. The column titles show the dependent variables. “Post” coefficients are multiplied by 1,000 to get the effect per 1,000 discharges. Baseline values show the average DKA per 1,000 discharges before the pandemic. Controls include patient characteristics, hospital and quarter dummies. % change calculated as Post/Baseline*100. *p*-values in parentheses. ***p < 0.01, **p < 0.05, *p < 0.1.

**Table 2:** Diabetic ketoacidosis mortality.

Variables	(1)	(2)	(3)	(4)	(5)
	All DKA	All DKA	All DKA	All DKA	Non-COVID DKA
Post	0.0132*** (0.0000)	0.0043** (0.0116)	0.0061*** (0.0000)	0.0029* (0.0743)	0.0065*** (0.0000)
COVID			(0.0000)	(0.0000)	
% Change	135%	44%	62%	30%	66%
Observations	83,983	83,983	83,983	83,983	80,753
Controls	Yes	Yes	Yes	Yes	Yes
Time-trend	No	Yes	No	Yes	No
Baseline	0.0098	0.0098	0.0098	0.0098	0.0098

Estimates from model 2. The dependent variable is the in-hospital mortality of patients with diabetic ketoacidosis (DKA). Column titles show inclusion criteria for DKA cases. “Post” is a dummy for the post-COVID period, “COVID” indicates the presence of COVID-19 in discharge records. Baseline values show the average mortality of patients with DKA in the pre-pandemic period. % change calculated as Post/Baseline*100. *p*-values in parentheses. *** *p* < 0.01, ** *p* < 0.05, * *p* < 0.1.

**Table 3:** Diabetic ketoacidosis length of hospital stay.

Variables	(1)	(2)	(3)	(4)	(5)
	All DKA	All DKA	All DKA	All DKA	Non-COVID DKA
Post	0.4203*** (0.0000)	−0.1188 (0.3327)	0.2056*** (0.0000)	−0.1598 (0.1896)	0.2268*** (0.0000)
COVID			2.5867*** (0.0000)	2.5344*** (0.0000)	
% Change	11%	−3%	5%	−4%	6%
Observations	83,979	83,979	83,979	83,979	80,749
Controls	Yes	Yes	Yes	Yes	Yes
Time trend	No	Yes	No	Yes	No
Baseline	3.91	3.92	3.92	3.92	3.92

Estimates from model 2. The dependent variable is the length of stay for patients with diabetic ketoacidosis (DKA). Column titles show inclusion criteria for DKA cases. “Post” is a dummy for the post-COVID period, “COVID” indicates the presence of COVID-19 in discharge records. Baseline values show the average length of hospital stays for patients with DKA in the pre-pandemic period. % change calculated as Post/Baseline*100. *p*-values in parentheses. *** *p* < 0.01, **p < 0.05, * *p* < 0.1.

Controlling for patient characteristics, hospital fixed effects, time trends, and quarterly fixed effects, the mortality rate for DKA patients increased by 0.43 percentage points, representing an approximately 44% increase compared to the pre-COVID period average mortality rate of 0.98%. When accounting for the COVID-19 comorbidity, the Post coefficient estimate decreases but still suggests a 30% increase from baseline levels. COVID-19 comorbidity is associated with a substantial increase in mortality. The estimated coefficients suggest 8-fold higher risk compared to pre-pandemic averages.

The most significant factor for the length of hospital stays is the COVID-19 comorbidity. Patients with COVID-19 stay in the hospital for approximately 2.5 days longer. This corresponds to a 64% increase compared to pre-COVID average length of stay for DKA patients. The results also suggest a slight increase in length of stay by about 0.2 days, which may be attributed to the indirect effects; however, this finding is not robust to the inclusion of a linear time trend, which reflects a pre-existing upward trajectory in LOS before the pandemic, as discussed in detail in the robustness section. Thus, the findings related to LOS from model 2 should be interpreted with caution.

### 3.2 Other acute emergencies: acute myocardial infarction and acute kidney injury

Figures A4 and A5 plot the raw average mortality and length of stay for discharges with AMI and AKI over time in comparison to DKA. Figure A4 reveals that both the mortality rate and the average length of hospital stays are significantly higher for patients with AMI than patients with DKA. However, in the post-COVID period, both outcomes deteriorated more dramatically for patients with DKA. Similarly, in the pre-COVID period, patients with AKI have higher mortality and longer hospital stays compared to patients with DKA as seen in Figure A5. After the pandemic, the mortality rate of patients with DKA increased at a faster rate and surpassed the average in-hospital mortality of patients with AKI.

**Figure 1:**
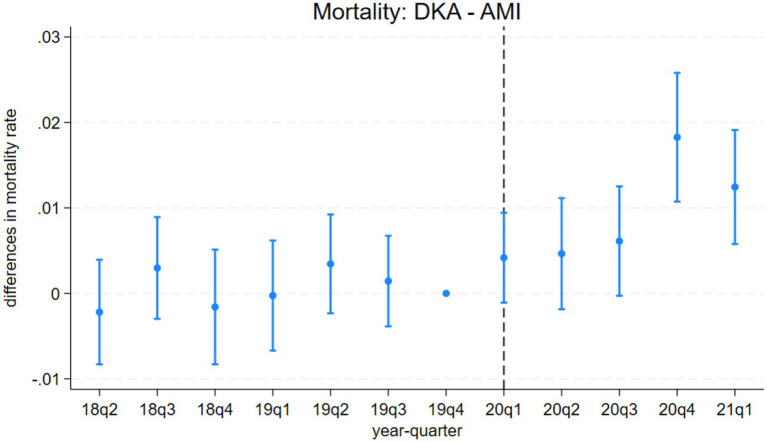
Differences in mortality rates between discharges with diabetic ketoacidosis (DKA) and acute myocardial infarction (AMI) over time. The coefficient estimates and 95% confidence intervals for the interactions of year-quarter dummies with DKA from model 3 are plotted. The dependent variable is an indicator for in-hospital mortality. Discharges with DKA and AMI are included. The last quarter of 2019 is the base period. The dashed line marks the beginning of the pandemic. Regressions include patient characteristics, year-quarter, and hospital dummies.

**Figure 2:**
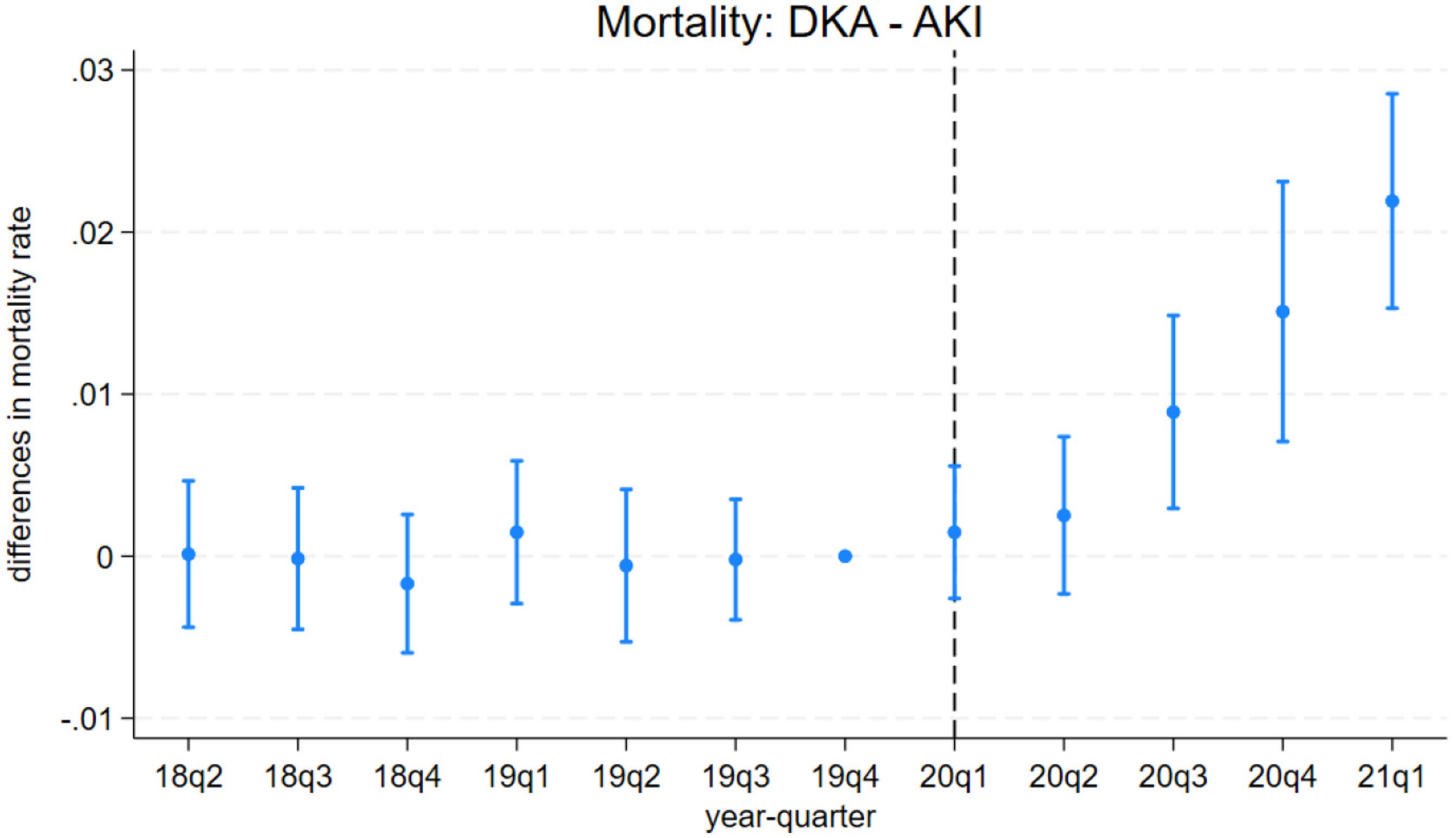
Differences in mortality rates between discharges with diabetic ketoacidosis (DKA) and acute kidney injury (AKI) over time. The coefficient estimates and 95% confidence intervals for the interactions of year-quarter dummies with DKA from model 3 are plotted. The dependent variable is an indicator for in-hospital mortality. Discharges with DKA and AKI are included. The last quarter of 2019 is the base period. The dashed line marks the beginning of the pandemic. Regressions include patient characteristics, year-quarter, and hospital dummies.

**Figure 3:**
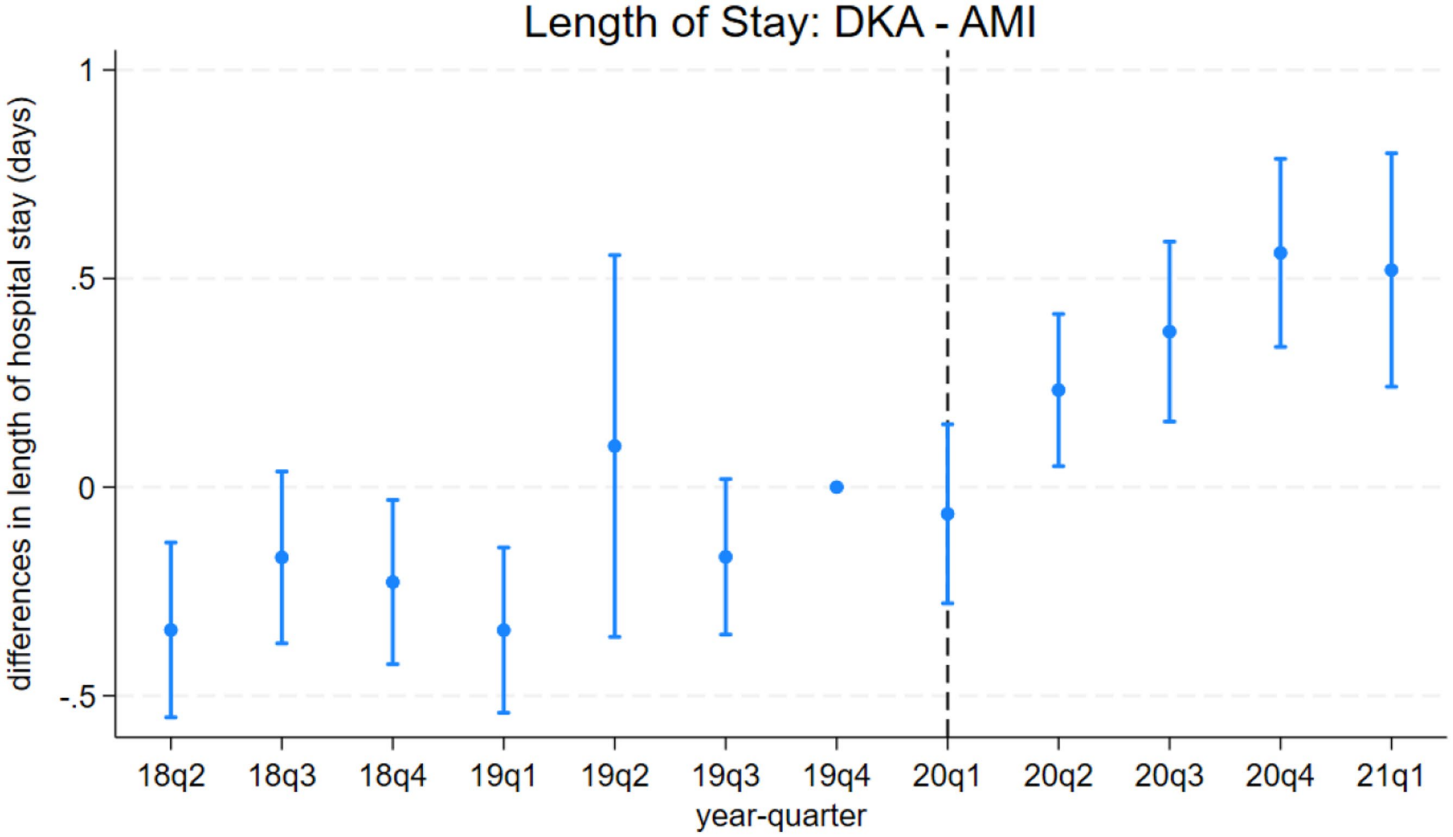
Differences in length of hospital stays between discharges with diabetic ketoacidosis (DKA) and acute myocardial infarction (AMI) over time. the coefficient estimates and 95% confidence intervals for the interactions of year-quarter dummies with DKA from model 3 are plotted. The dependent variable is the length of hospital stays (days). Discharges with DKA and AMI are included. The last quarter of 2019 is the base period. The dashed line marks the beginning of the pandemic. Regressions include patient characteristics, year-quarter, and hospital dummies.

**Figure 4:**
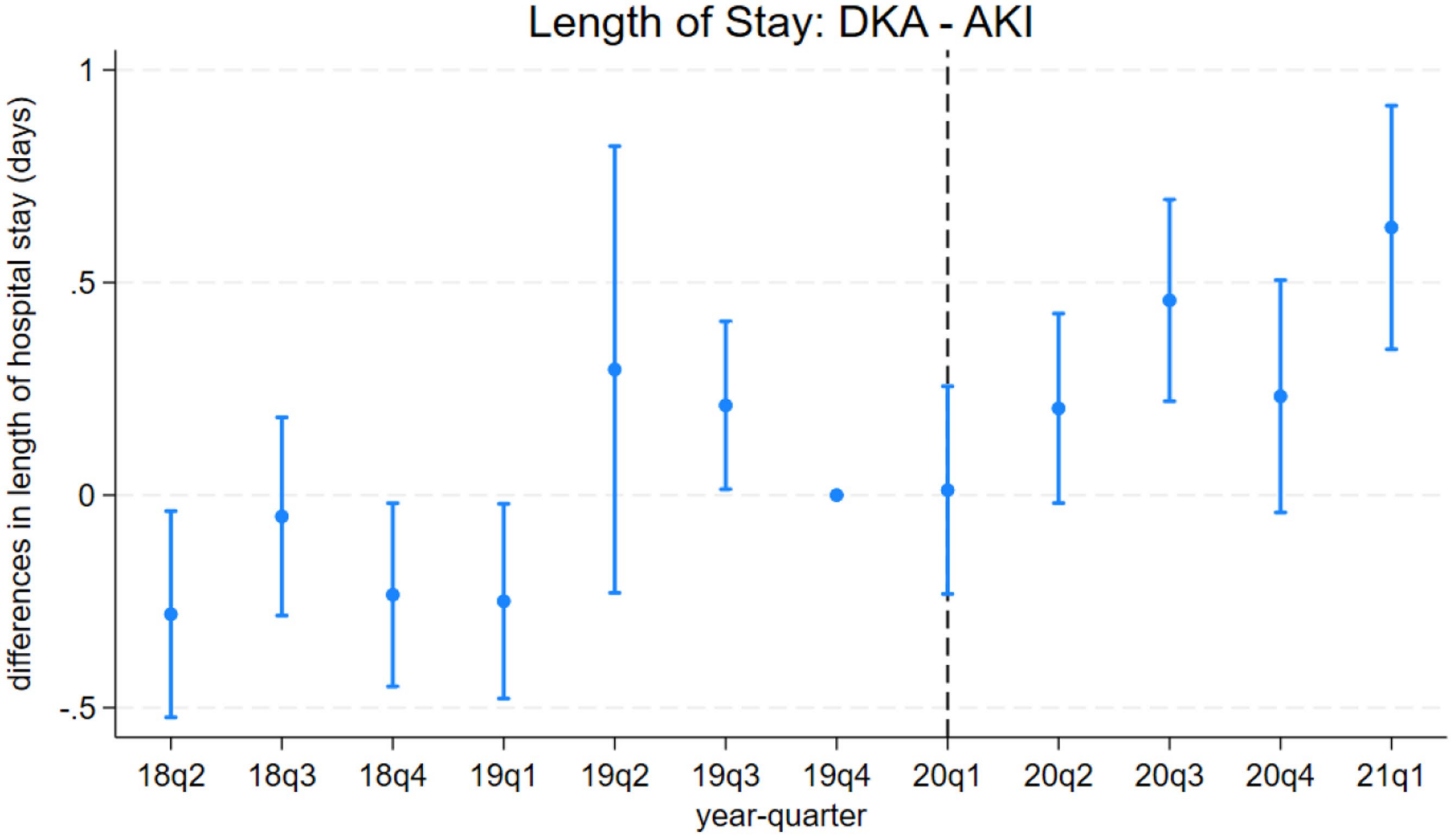
Differences in length of hospital stays between discharges with diabetic ketoacidosis (DKA) and acute kidney injury (AKI) over time. The coefficient estimates and 95% confidence intervals for the interactions of year-quarter dummies with DKA from model 3 are plotted. The dependent variable is the length of hospital stays (days). Discharges with DKA and AKI are included. The last quarter of 2019 is the base period. The dashed line marks the beginning of the pandemic. Regressions include patient characteristics, year-quarter, and hospital dummies.

The event study results from model 3 are presented in Figures 1–4. Figure 1 indicates a differential impact of the COVID-19 pandemic on mortality rates between patients with DKA and those with AMI. By late 2020, the average mortality rate among DKA patients was almost two percentage points higher compared to AMI patients. The event study framework further allows us to test for any pre-existing trends in mortality differences between these two patient populations. As depicted in Figure 1, the mortality rate difference remained stable before the pandemic, but a significant divergence occurred afterward. Consistent with these findings, Figure 2 demonstrates that the pandemic’s impact on DKA patient mortality exceeded that observed among AKI patients. Additionally, the figure confirms the absence of a significant pre-pandemic trend, as mortality differences between these groups were statistically indistinguishable from zero prior to the pandemic.

Model 4 provides a more detailed analysis of the pandemic’s differential impact on DKA patients, considering various mediating factors. The results, summarized in Tables 4 and 5, show that while COVID-19 comorbidity substantially increases mortality across all patient groups, the effect is 4.3 percentage points lower for DKA patients compared to AMI patients and 5.5 percentage points lower compared to AKI patients. Specifically, the model estimates an 8.5 percentage point increase in DKA mortality due to COVID-19 comorbidity, corresponding to more than an 8-fold increase from the pre-pandemic average. Moreover, the mortality rate among DKA patients is significantly affected more by indirect pandemic-related factors than that of AMI and AKI patients. The estimated differential impacts for DKA patients compared to AMI and AKI patients are 0.67 percentage points and 0.61 percentage points, respectively, representing a 62–68% increase from the baseline mortality rate.

**Table 4:** Differences in outcomes: diabetic ketoacidosis vs. acute myocardial infarction.

Variables	(1)	(2)	(3)	(4)
	Mortality	Mortality	Length of Stay	Length of Stay
Post	−0.0012 (0.5134)	−0.0017 (0.3375)	−0.2897*** (0.0000)	−0.2759*** (0.0000)
DKA	−0.0204*** (0.0000)	−0.0213*** (0.0000)	−0.0892 (0.2408)	−0.1166 (0.1259)
DKA*Post	0.0091*** (0.0000)	0.0066*** (0.0000)	0.4964*** (0.0000)	0.3503*** (0.0000)
COVID		0.1280*** (0.0000)		2.1342*** (0.0000)
DKA*COVID		−0.0429*** (0.0001)		0.5229** (0.0172)
Observations	291,111	291,111	291,084	291,084
DKA*Pre-COVID Trend	0.0004 (0.4035)	0.0004 (0.4035)	0.0509*** (0.0026)	0.0509*** (0.0026)

Estimates from model 4. Discharges with diabetic ketoacidosis (DKA) or acute myocardial infarction (AMI) are included. Column titles show dependent variables. “Post” is a dummy for the post-COVID period, “COVID” indicates the presence of COVID-19 comorbidity. All regressions include patient characteristics, hospital and quarter dummies, and a time trend. The interaction of the time trend in the pre-COVID period with DKA is included to provide a test for parallel trends assumption. Robust *p*-values in parentheses. *** *p* < 0.01, ** *p* < 0.05, * *p* < 0.1.

**Table 5:** Differences in outcomes: diabetic ketoacidosis vs. acute kidney injury.

Variables	(1)	(2)	(3)	(4)
	Mortality	Mortality	Length of Stay	Length of Stay
Post	0.0032** (0.0109)	0.0023** (0.0305)	0.0151 (0.7857)	−0.0059 (0.9113)
DKA	0.0052*** (0.0000)	0.0048*** (0.0000)	−0.4452*** (0.0013)	−0.4582*** (0.0010)
DKA*Post	0.0111*** (0.0000)	0.0061*** (0.0000)	0.3801*** (0.0000)	0.2224*** (0.0015)
COVID		0.1410*** (0.0000)		3.6883*** (0.0000)
DKA*COVID		−0.0553** (0.0129)		−1.1610*** (0.0093)
Observations	429,330	429,330	429,284	429,284
DKA*Pre-COVID Trend	0.0001 (0.7973)	0.0001 (0.7973)	0.0714*** (0.0005)	0.0714*** (0.0005)

Estimates from model 4. Discharges with diabetic ketoacidosis (DKA) or acute kidney injury (AKI) are included. The column titles show dependent variables. “Post” is a dummy for the post-COVID period, “COVID” indicates the presence of COVID-19 comorbidity. All regressions include patient characteristics, hospital and quarter dummies, and a time trend. The interaction of the time trend in the pre-COVID period with DKA is included to provide a test for parallel trends assumption. Robust p-values in parentheses. *** *p* < 0.01, ** *p* < 0.05, * *p* < 0.1.

LOS results from model 4 align with our mortality findings regarding the pandemic’s indirect effects. DKA patients without COVID-19 comorbidity experienced a hospital stay approximately 0.4 days longer than AMI and AKI patients. Although a significant increase in LOS is observed among COVID-19 patients, the differential effect of COVID-19 comorbidity on LOS for DKA patients remains inconclusive. While the relative post-pandemic increase in length of stay for DKA is statistically significant, visual analysis of Figures 3, 4 suggests the presence of a pre-pandemic trend. This trend may indicate that the observed post-pandemic increase in LOS is a continuation of an existing pre-pandemic pattern.

### 3.3 Robustness analyses

One limitation of our analysis arises from the fact that we do not have a proper comparison group that can capture already existing pre-pandemic trends. This limitation is particularly relevant to the potential collinearity between COVID status and time trends, as the lack of a comparison group makes it more difficult to isolate the effects of the pandemic from pre-existing trends in the outcome variables. To address this, we included a linear time trend in our models to account for underlying patterns that may have existed before the pandemic. This approach helps evaluate whether our findings are driven by the pandemic or by a pre-existing linear trend. Given the relatively short pre-pandemic period in our dataset, adding a time trend captures some of the true pandemic effects while accounting for pre-existing trends. Thus, adding the time trend gives a more conservative estimate of the pandemic’s impact. On the other hand, this specification helps mitigate concerns about potential collinearity between COVID status and time trends. Since COVID-19 diagnoses are observed only in the post-pandemic period, there is a structural overlap between COVID status, the Post variable that captures the indirect pandemic effects and the timing of observations. By including a linear time trend in the model, we reduce the risk that the effects attributed to COVID status or the indirect pandemic effects are driven by the pre-existing trends. Results with the time trend are presented in Tables 1–3. Incorporating the time trend, we observe that the increase in DKA frequency and mortality results remain significantly positive, suggesting a robust increase in DKA cases and mortality rates post-pandemic. However, LOS results lose statistical significance and even change direction, reflecting the pre-existing upward trend in LOS for DKA patients prior to the pandemic, which is also visible in Figure A2 panel 3. These results strengthen confidence in the robustness of DKA frequency and mortality results, though findings for LOS should be interpreted with caution.

As another robustness check, we restricted the post-pandemic sample to DKA discharges without documented COVID-19 comorbidity. As explained in the methodology section, this approach removes the direct viral effects on outcomes, which are absent by definition in the pre-pandemic period. However, COVID-19 incidence and mortality rates were disproportionately higher among certain subgroups, such as African American and Hispanic people, who on average have higher uninsured rates, thus lower access to healthcare (37). Additionally, as COVID-positive patients may constitute a higher-risk group even in the absence of infection, their exclusion in the pandemic period could lead to a downward bias in estimating the impact of pandemic-related healthcare disruptions (36). Thus, this analysis offers a complementary, conservative estimate of the pandemic’s indirect effects. Our estimates from this restricted sample show consistent results, with coefficient estimates similar to those found in our preferred model for mortality and LOS outcomes, further reinforcing the robustness of our findings.

We analyzed pre-pandemic trends by comparing DKA patients with AMI and AKI patients separately to check for any pre-existing differential trend in outcomes. The results of these differential trend tests are presented in Tables 4 and 5 for AMI and AKI patients, respectively. For mortality, the coefficient for the linear differential trend between DKA and AMI patients is quite small and not statistically significant (0.0004, *p*-value = 0.4064). However, for LOS, we observe a substantial and statistically significant trend difference during the pre-pandemic period (0.0507, p-value = 0.0028). A similar pattern emerges when comparing DKA and AKI patients. Regarding mortality, the linear differential trend coefficient is minimal and not statistically significant (0.0001, p-value = 0.8255), yet the trend difference between groups for LOS is larger and statistically significant (0.0709, p-value = 0.0005). These findings are consistent with our descriptive trend analysis, which indicates a pre-pandemic upward trend in LOS for patients with DKA. Therefore, while our results for the mortality outcome remain robust, the findings related to LOS should be interpreted with caution due to these pre-existing trends.

Since non-urgent hospitalizations abruptly dropped during COVID-19, using a measure of per 1,000 discharges may inflate the increase in DKA frequencies. The dataset includes information on the type of admissions (categorized as Emergency, Urgent, Elective, Newborn, Trauma, or Unknown). More than 96% of DKA admissions are classified as emergency or urgent, while elective procedures were halted at the beginning of the pandemic. As a robustness check, we repeated the analyses focusing solely on emergency and urgent admissions. The results in Table 6 still show a significant increase in DKA discharges during the pandemic period. DKA frequencies increased by 10–20%. Additionally, there was a substantial rise in non-COVID DKA cases, suggesting that pandemic-related disruptions played a significant role.

**Table 6:** Diabetic ketoacidosis frequency (emergency and urgent admissions only).

Variables	(1)	(2)	(3)	(4)	(5)	(6)
	DKA	DKA	DKA	Non-COVID DKA	Non-COVID DKA	Non-COVID DKA
Post	2.65*** (0.0000)	2.47*** (0.0000)	1.39*** (0.0000)	1.27*** (0.0000)	1.13*** (0.0000)	1.09*** (0.0003)
Observations	5,565,618	5,565,618	5,565,618	5,565,618	5,565,618	5,565,618
Controls	No	Yes	Yes	No	Yes	Yes
Time-trend	No	No	Yes	No	No	Yes
Baseline	13.4	13.4	13.4	13.4	13.4	13.4

Estimates from model 1. The sample is restricted to discharge records where the type of admission is classified as either emergency or urgent. Column titles show the dependent variables. “Post” coefficients are multiplied by 1,000 to get the effect per 1,000 discharges. Baseline values show the average DKA per 1,000 discharges before the pandemic. Controls include patient characteristics, hospital and quarter dummies. % change calculated as Post/Baseline*100. *p*-values in parentheses. *** *p* < 0.01, ** *p* < 0.05.

To address potential inaccuracies in COVID-19 diagnoses during the early stages of the pandemic, when testing may not have been widely or uniformly implemented, we conducted an additional robustness check by excluding data from the first two quarters of 2020. This approach also addresses the possibility of under-coding COVID-19 cases early in the pandemic, which could affect the validity of our estimated indirect effects. The results from this analysis, shown in Tables 7–9, support the validity of our findings. The estimates for the COVID coefficient are consistent across models. The coefficient for the post-pandemic variable in our preferred model is comparable (0.009 compared to 0.006) and statistically significant at the 1% level, highlighting the robustness of the observed increase in mortality rate post-pandemic that we attribute to disruptions in the healthcare system. Incorporating the time trend into model 2 (column 4,) the post-pandemic coefficient becomes larger and statistically significant at the 1% level. These results support that the increased mortality post-pandemic is not attributable to early misclassification of COVID-19 diagnoses, providing evidence for our findings’ robustness. The robustness analysis results regarding the frequency of hospitalizations are also consistent with our earlier findings. The coefficient for the post-pandemic variable increases to 2.07 additional DKA hospitalizations per 1,000 discharges, compared to 1.74 in the original model. These findings further support the robustness of our results showing an increase in hospitalization frequency post-pandemic. The results from model 4, reported in Tables 10 and 11, are also very similar.

**Table 7:** Diabetic ketoacidosis frequency (2020 q1 and q2 dropped).

Variables	(1)	(2)	(3)	(4)	(5)	(6)
	DKA	DKA	DKA	Non-COVID DKA	Non-COVID DKA	Non-COVID DKA
Post	2.40*** (0.0000)	2.07*** (0.0000)	1.77*** (0.0000)	0.96*** (0.0000)	0.68*** (0.0000)	0.54** (0.0437)
Observations	7,256,415	7,256,415	7,256,415	7,256,415	7,256,415	7,256,415
Controls	No	Yes	Yes	No	Yes	Yes
Time-trend	No	No	Yes	No	No	Yes
Baseline	8.95	8.95	8.95	8.95	8.95	8.95

The first and second quarters of 2020 are excluded from the analysis. Estimates from model 1. Column titles show the dependent variables. “Post” coefficients are multiplied by 1,000 to get the effect per 1,000 discharges. Baseline values show the average DKA per 1,000 discharges before the pandemic. Controls include patient characteristics, hospital and quarter dummies. *p* < 0.01, ** *p* < 0.05, * *p* < 0.1.

**Table 8:** Diabetic ketoacidosis mortality (2020 q1 and q2 dropped).

Variables	(1)	(2)	(3)	(4)	(5)
	All DKA	All DKA	All DKA	All DKA	Non-COVID DKA
Post	0.0194*** (0.0000)	0.0206*** (0.0000)	0.0091*** (0.0000)	0.0113*** (0.0000)	0.0094*** (0.0000)
COVID			0.0844*** (0.0000)	0.0844*** (0.0000)	
Observations	69,969	69,969	69,969	69,969	66,931
Controls	Yes	Yes	Yes	Yes	Yes
Time-trend	No	Yes	No	Yes	No
Baseline	0.0098	0.0098	0.0098	0.0098	0.0098

The first and second quarters of 2020 are excluded from the analysis. Estimates from model 2. The dependent variable is the in-hospital mortality of patients with DKA. The column titles show inclusion criteria for DKA cases. “Post” is a dummy for the post-COVID period, “COVID” indicates the presence of COVID-19 in discharge records. The baseline values show the average mortality of patients with DKA in the pre-pandemic period. *p*-values in parentheses. *** *p* < 0.01, ** *p* < 0.05, * *p* < 0.1.

**Table 9:** Diabetic ketoacidosis length of hospital stay (2020 q1 and q2 dropped).

Variables	(1)	(2)	(3)	(4)	(5)
	All DKA	All DKA	All DKA	All DKA	Non-COVID DKA
Post	0.6405*** (0.0000)	0.2836* (0.0764)	0.3279*** (0.0000)	0.0020 (0.9900)	0.3398*** (0.0000)
COVID			2.5723*** (0.0000)	2.5664*** (0.0000)	
Observations	69,969	69,969	69,969	69,969	66,931
Controls	Yes	Yes	Yes	Yes	Yes
Time trend	No	Yes	No	Yes	No
Baseline	3.91	3.92	3.92	3.92	3.92

The first and second quarters of 2020 are excluded from the analysis. Estimates from model 2. The dependent variable is the length of stay for patients with DKA. The column titles show inclusion criteria for DKA cases. “Post” is a dummy for the post-COVID period, “COVID” indicates the presence of COVID-19 in discharge records. Baseline values show the average length of hospital stays for patients with DKA in the pre-pandemic period. *p*-values in parentheses *** *p* < 0.01, ** *p* < 0.05, * *p* < 0.1.

**Table 10:** Differences in outcomes: diabetic ketoacidosis vs. acute myocardial infarction (2020 q1 and q2 dropped).

Variables	(1)	(2)	(3)	(4)
	Mortality	Mortality	Length of Stay	Length of Stay
Post	0.0101*** (0.0000)	0.0038* (0.0609)	−0.1057 (0.1579)	−0.2097*** (0.0050)
DKA	−0.0197*** (0.0000)	−0.0207*** (0.0000)	−0.0850 (0.2630)	−0.1157 (0.1280)
DKA*Post	0.0117*** (0.0000)	0.0081*** (0.0000)	0.6495*** (0.0000)	0.4367*** (0.0000)
COVID		0.1275*** (0.0000)		2.2119*** (0.0000)
DKA*COVID		−0.0426*** (0.0001)		0.4501** (0.0496)
Observations	242,841	242,841	242,841	242,841

The first and second quarters of 2020 are dropped. Estimates from model 4. Discharges with DKA or AMI are included. Column titles show dependent variables. “Post” is a dummy for the post-COVID period, “COVID” indicates the presence of COVID-19 comorbidity. All regressions include patient characteristics, hospital and quarter dummies, and a time trend. Robust p*-*values in parentheses *** *p* < 0.01, ** *p* < 0.05, * *p* < 0.1.

**Table 11:** Differences in outcomes: diabetic ketoacidosis vs. acute kidney injury (2020 q1 and q2 dropped).

Variables	(1)	(2)	(3)	(4)
	Mortality	Mortality	Length of Stay	Length of Stay
Post	0.0113*** (0.0000)	0.0061*** (0.0000)	0.2977*** (0.0001)	0.1626** (0.0151)
DKA	0.0053*** (0.0000)	0.0048***	−0.4398*** (0.0021)	−0.4542*** (0.0015)
		(0.0000)		
DKA*Post	0.0157*** (0.0000)	0.0088*** (0.0000)	0.4935*** (0.0000)	0.2700*** (0.0027)
COVID		0.1443*** (0.0000)		3.7726*** (0.0000)
DKA*COVID		−0.0594*** (0.0098)		−1.2655*** (0.0059)
Observations	364,713	364,713	364,713	364,713

The first and second quarters of 2020 are dropped. Estimates are from model 4. Discharges with DKA or AKI are included. Column titles show dependent variables. “Post” is a dummy for the post-COVID period, “COVID” indicates the presence of COVID-19 comorbidity. All regressions include patient characteristics, hospital and quarter dummies, and a time trend. Robust *p*-values in parentheses. *** *p* < 0.01, ** *p* < 0.05, * *p* < 0.1.

To further assess the robustness of our findings, we replace the COVID-19 comorbidity indicator variable with the Charlson Comorbidity Index (CCI), which predicts patient mortality risk based on the presence of comorbid conditions. This adjustment addresses the limitation that the COVID-19 comorbidity variable is only measurable in the post-pandemic period. By introducing an index that consistently captures the risk profile of all patients across the entire study period, we ensure a more uniform basis for comparison. The results using CCI, presented in Table 12, remain consistent with our primary findings. The coefficient for the post-pandemic variable in model 2 shows a significant increase in mortality after controlling for patient risk using CCI, further supporting the robustness of our results. Similarly, replacing the COVID-19 indicator variable with CCI in model 4 produces consistent results regarding the differential impact of the COVID-19 period on mortality among DKA patients compared to AMI and AKI patients. These findings suggest that our results are not driven by changes in the availability or measurement of the COVID-19 comorbidity variable but instead reflect broader disruptions in the healthcare system during the pandemic.

**Table 12:** Change in mortality (CCI instead of COVID-19).

Variables	DKA Only		DKA and AMI		DKA and AKI	
	(1)	(2)	(3)	(4)	(5)	(6)
Post	0.0130*** (0.0000)	0.0044*** (0.0097)	0.0035** (0.0114)	−0.0013 (0.4644)	0.0058*** (0.0000)	0.0030** (0.0186)
DKA			−0.0179*** (0.0000)	−0.0178*** (0.0000)	0.0050*** (0.0000)	0.0050*** (0.0000)
DKA*Post			0.0095*** (0.0000)	0.0094*** (0.0000)	0.0113*** (0.0000)	0.0112*** (0.0000)
Observations	83,983	83,983	291,111	291,111	429,330	429,330
Controls	Yes	Yes	Yes	Yes	Yes	Yes
Time-trend	No	Yes	No	Yes	No	Yes
Baseline	0.0098	0.0098	0.0098	0.0098	0.0098	0.0098

The dependent variable is the in-hospital mortality. Charlson Comorbidity Index (CCI) is used instead of COVID-19 dummy to control for mortality risk. The column titles show inclusion criteria for discharges. “Post” is a dummy for the post-COVID period, “DKA” is an indicator for discharges with diabetic ketoacidosis. Baseline values show the average mortality of patients with DKA in the pre-pandemic period. *p*-values in parentheses. *** *p* < 0.01, ** *p* < 0.05, * *p* < 0.1.

Our data set includes patient zip codes, which may be used as proxies for socioeconomic and geographic confounders. Since more than 10% of the data is missing zip code, we did not include it in our main analysis to avoid any bias. However, we conduct another robustness check by including dummies for patients’ zip codes among the control variables. The results, reported in Tables 13–17, are consistent with our earlier findings.

**Table 13:** Diabetic ketoacidosis frequency (control for patient zip codes).

Variables	(1)	(2)	(3)	(4)	(5)	(6)
	DKA	DKA	DKA	Non-COVID DKA	Non-COVID DKA	Non-COVID DKA
Post	1.98*** (0.0000)	01.52*** (0.0000)	0.60*** (0.0000)	1.05*** (0.0000)	0.59*** (0.0000)	0.40*** (0.0038)
Observations	8,622,092	7,899,656	7,899,656	8,622,092	7,899,656	7,899,656
Controls	No	Yes	Yes	No	Yes	Yes
Time-trend	No	No	Yes	No	No	Yes
Baseline	8.95	8.95	8.95	8.95	8.95	8.95

Estimates from model 1. The column titles show the dependent variables. “Post” coefficients are multiplied by 1,000 to get the effect per 1,000 discharges. Baseline values show the average DKA per 1,000 discharges before the pandemic. Controls include patient characteristics, patient zip codes and quarter dummies. *p*-values in parentheses. *** *p* < 0.01, ** *p* < 0.05, * *p* < 0.1.

**Table 14:** Diabetic ketoacidosis mortality (control for patient zip codes).

Variables	(1)	(2)	(3)	(4)	(5)
	All DKA	All DKA	All DKA	All DKA	Non-COVID DKA
Post	0.0147*** (0.0000)	0.0051** (0.0177)	0.0068*** (0.0000)	0.0035* (0.0940)	0.0073*** (0.0000)
COVID			0.0869*** (0.0000)	0.0864*** (0.0000)	
Observations	71,707	71,707	71,707	71,707	68,677
Controls	Yes	Yes	Yes	Yes	Yes
Time-trend	No	Yes	No	Yes	No
Baseline	0.0098	0.0098	0.0098	0.0098	0.0098

Estimates from model 2. The dependent variable is the in-hospital mortality of patients with diabetic ketoacidosis (DKA). Column titles show inclusion criteria for DKA cases. “Post” is a dummy for the post-COVID period, “COVID” indicates the presence of COVID-19 in discharge records. Baseline values show the average mortality of patients with DKA in the pre-pandemic period. Controls include patient characteristics, patient zip codes and quarter dummies. *p*-values in parentheses. *** *p* < 0.01, ** *p* < 0.05, * *p* < 0.1.

**Table 15:** Diabetic ketoacidosis length of hospital stay (control for patient zip codes).

Variables	(1)	(2)	(3)	(4)	(5)
	All DKA	All DKA	All DKA	All DKA	Non-COVID DKA
Post	0.4357*** (0.0000)	−0.0838 (0.4323)	0.2070*** (0.0001)	−0.1272 (0.2323)	0.2339*** (0.0000)
COVID			2.5233*** (0.0000)	2.4756*** (0.0000)	
Observations	71,703	71,703	71,703	71,703	68,673
Controls	Yes	Yes	Yes	Yes	Yes
Time trend	No	Yes	No	Yes	No
Baseline	3.91	3.92	3.92	3.92	3.92

Estimates from model 2. The dependent variable is the length of hospital stay for patients with diabetic ketoacidosis (DKA). Column titles show inclusion criteria for DKA cases. “Post” is a dummy for the post-COVID period, “COVID” indicates the presence of COVID-19 in discharge records. Baseline values show the average length of hospital stays for patients with DKA in the pre-pandemic period. Controls include patient characteristics, patient zip codes and quarter dummies. *p*-values in parentheses. *p*-values in parentheses. *** *p* < 0.01, **p < 0.05, * *p* < 0.1.

**Table 16:** Differences in outcomes: diabetic ketoacidosis vs. acute myocardial infarction (control for patient zip codes).

Variables	(1)	(2)	(3)	(4)
	Mortality	Mortality	Length of Stay	Length of Stay
Post	−0.0009 (0.6265)	−0.0014 (0.4226)	−0.2922*** (0.0000)	−0.2765*** (0.0000)
DKA	−0.0201*** (0.0000)	−0.0211*** (0.0000)	−0.2487*** (0.0000)	−0.2785*** (0.0000)
DKA*Post	0.0105*** (0.0000)	0.0074*** (0.0001)	0.5494*** (0.0000)	0.3841*** (0.0000)
COVID		0.1303*** (0.0000)		2.0669*** (0.0000)
DKA*COVID		−0.0434*** (0.0000)		0.5546*** (0.0007)
Observations	262,360	262,360	262,333	262,333

Estimates from model 4. Discharges with diabetic ketoacidosis (DKA) or acute myocardial infarction (AMI) are included. Column titles show dependent variables. “Post” is a dummy for the post-COVID period, “COVID” indicates the presence of COVID-19 comorbidity. All regressions include patient characteristics, patient zip codes and quarter dummies, and a time trend. Robust *p*-values in parentheses. *** *p* < 0.01, ** *p* < 0.05, * *p* < 0.1.

**Table 17:** Differences in outcomes: diabetic ketoacidosis vs. acute kidney injury (control for patient zip codes).

Variables	(1)	(2)	(3)	(4)
	Mortality	Mortality	Length of Stay	Length of Stay
Post	0.0038*** (0.0001)	0.0028*** (0.0049)	0.0252 (0.5710)	0.0050 (0.9107)
DKA	0.0059*** (0.0000)	0.0054*** (0.0000)	−0.7192*** (0.0000)	−0.7345*** (0.0000)
DKA*Post	0.0130*** (0.0000)	0.0075*** (0.0000)	0.3952*** (0.0000)	0.2106*** (0.0004)
COVID		0.1466*** (0.0000)		3.6096*** (0.0000)
DKA*COVID		−0.0603*** (0.0000)		−1.0479*** (0.0000)
Observations	384,700	384,700	384,658	384,658

Estimates from model 4. Discharges with diabetic ketoacidosis (DKA) or acute kidney injury (AKI) are included. The column titles show dependent variables. “Post” is a dummy for the post-COVID period, “COVID” indicates the presence of COVID-19 comorbidity. All regressions include patient characteristics, patient zip codes and quarter dummies, and a time trend. Robust *p*-values in parentheses. *** *p* < 0.01, ** *p* < 0.05, * *p* < 0.1.

## 4 Discussion

Our analysis shows a significant rise in DKA hospitalizations during the pandemic, with a significant part of the increase driven by cases without COVID-19 comorbidity. After adjusting for patient characteristics and other factors, DKA discharges remained elevated. We associate this result with various disruptions in the healthcare system, such as delayed care, limited access to primary care or strain on hospital resources. Mortality rates for DKA patients also showed a significant increase, and COVID-19 comorbidity further exacerbated this risk. Additionally, COVID-19 comorbidity was associated with notably longer hospital stays for DKA patients compared to the pre-pandemic level, but our analysis regarding the LOS is not conclusive due to the pre-existing upward trend in the data. It is important to note that some policies, such as expanded telehealth services or emergency prescription dispensing rules during the COVID-19 pandemic, may have mitigated the pandemic’s indirect effects, and thus the findings of the current study reflect the net effects.

Comparatively, mortality outcomes were disproportionately affected among patients with DKA compared to patients with AMI and AKI during the pandemic despite similar pre-pandemic trends among the conditions. We used event-study and difference-in-differences models with hospital and time fixed effects beyond descriptive comparisons. This allows us to robustly assess whether DKA patients were disproportionately affected by the pandemic relative to AMI and AKI patients, while also providing a structured check on pre-pandemic trend differences. Our findings also suggest that the pandemic’s indirect effects impacted patients with DKA more relative to AMI and AKI cases. While COVID-19 comorbidity significantly influenced mortality across all patient groups, the pandemic’s indirect consequences seem to have particularly intensified adverse outcomes for patients with DKA.

The current study underlines the role of non-viral channels including delays in seeking or receiving care, reduced access to routine outpatient services, and strains on hospital capacity on DKA mortality. While the goal of this study is not to disentangle these channels, the comparison analysis with AKI and AMI may suggest delays in care and issues with glycemic control are the main indirect mechanisms affecting DKA mortality disproportionately. Assuming patients treated within the same hospitals would be similarly affected by hospital-level constraints and shortages, any excess mortality increase in DKA is presumably associated with reduced access to routine outpatient services, delayed care and poor glycemic control. Table A2 which shows mortality risk for DKA, AMI, and AKI patients before and after the pandemic also supports this hypothesis by showing that the increase in the mortality risk of patients with DKA is higher than that of AMI and AKI. Since DKA requires longitudinal care for prevention, any disruptions in primary care access and continuity disproportionately increases its prevalence and severity.

Increased DKA hospitalizations and higher mortality rates during the pandemic also have important economic implications. Increased hospitalizations add direct costs in terms of hospital bed-days, nursing effort, and pharmacy use. Higher mortality rate among DKA hospitalizations implies increased intensive care use and resource-intensive management. Our results related to DKA patients being disproportionately affected by the indirect channels compared to patients with AMI or AKI suggest that delays in diabetes care and glycemic management may be especially costly drivers of pandemic-related burden. Beyond hospital expenditures, these forces may have crowded out capacity for other acute conditions during COVID-19 surges. Thus, policies that reduce DKA admissions or mitigate severity at admission have both clinical and economic value, as they may reduce direct hospital costs and preserve system-wide capacity during future health crises.

Several policy recommendations can help reduce the incidence of diabetic ketoacidosis (DKA) and improve diabetes care in both routine healthcare settings and during public health emergencies. Ensuring uninterrupted access to essential medications, especially insulin, is critical. The World Health Organization’s initiative to provide emergency health kits aimed to provide relief to 10.000 people for approximately 3 months represents a potential approach that could help address challenges such in insulin access. Interagency Emergency Health Kit 2024 included basic diabetes care resources such as insulin, glucometer and supplies. Similar initiatives can be adopted by states or governments or local institutions to provide at least one-time emergency aid. This may also require financial preparedness and advance planning for possible emergency situations. Other policies to improve insulin access may include insurance coverage expansion, price caps, extended 90-day medication supplies, or emergency refill protocols implemented by providers or institutions. The Centers for Medicare & Medicaid Services’ cap for out-of-pocket insulin costs at $35/month for Medicare beneficiaries is an example of a federal-level initiative to increase insulin access by reducing the cost (38). Another example is California’s CalRx program for reducing insulin cost by public production of biosimilar insulin (39). Partnerships with pharmaceutical companies and charitable organizations can further support low-income patients in accessing lifesaving treatments.

Expanding telemedicine access and adoption can support monitoring medication compliance and timely therapy adjustments. A hospital in Singapore has rapidly transitioned to a telehealth strategy, managing diabetes through virtual consultations and remote monitoring, which might be a safe and effective way in maintaining glycemic control and reducing hypoglycemia risk during a pandemic (40). Similarly, virtual diabetes clinics were employed in the UK during COVID 19 pandemic, providing an alternative strategy for type 2 diabetes management and highlighting the potential role of modern care delivery methods (41). Although telemedicine offers a flexible and scalable platform for diabetes care, particularly valuable during health crises, barriers such as limited internet access, low digital literacy, and lack of access to devices need to be addressed. Solutions may include developing user-friendly, accessible platforms and providing targeted digital education for patients with limited technological experience.

Studies over time consistently demonstrated that the use of continuous glucose monitor (CGM) technologies improve glycemic control in patients with diabetes (42). When integrated into telemedicine practice, CGM use can provide crucial information that can lead to more accurate analysis and eventually lead to superior outcomes (43). Improving the affordability and accessibility of remote monitoring tools is equally important in both stable and emergency conditions.

Strengthening the outpatient diabetes management infrastructure to function effectively during times of crisis is vital. Facilitating universal availability of Diabetes Self-Management Education (DSME), with a focus on high-risk individuals (e.g., those with prior DKA admissions), and developing an emergency readiness plan tailored to diabetes care can promote both long-term disease control and preparedness for future crises.

Evidence from other states and countries indicates that increases in DKA cases during the pandemic were widespread, and not unique to Texas. A descriptive study from New York hospitals reported that the prevalence of DKA nearly quadrupled during the pandemic compared to pre-pandemic periods (4). In England, researchers found higher DKA incidence among both patients with pre-existing type 2 diabetes and newly diagnosed diabetes, as well as changes in the characteristics of presenting cases (44). German registry data also documented increases in both type 1 diabetes incidence and DKA at presentation (45). A study using data from Africa (Tunisia) found a significant rise in DKA incidence during the pandemic (46).

While studies attempting to differentiate channels that affect patients with diabetes during the pandemic is sparse, the available evidence aligns closely with our findings. Using death certificate data from the National Vital Statistics System, a study from the US found excess mortality of 36.9% in 2020 and 46.6% in 2021 related to DKA after accounting for long-term mortality trends (23). This closely matches our own model 2 estimate of a 44% increase in DKA-related mortality after controlling for pre-existing time trends. The study also found that only a portion of these excess deaths were directly attributable to COVID-19 infection—51.3% in 2020 and 63.4% in 2021, which suggests pandemic-related healthcare disruptions, including delayed care and behavioral stressors, played a significant role. An international multicenter study based on data from 13 national diabetes registries found that the prevalence of DKA at the diagnosis of type 1 diabetes in children increased significantly during the COVID-19 pandemic. The increase was linked to pandemic containment measures not to the severity of COVID-19 (47).

Taken together, while Texas-specific structural features (high uninsured rates, rural healthcare constraints, and relatively mild COVID-19 restrictions) may influence the magnitude of the observed effects, the overall direction of changes in DKA incidence and mortality is consistent with findings from other settings, and indirect factors are also likely to play a significant role beyond Texas.

## 5 Limitations

One limitation of the analysis comparing DKA to other acute conditions stems from the fact that the hospitalization data are not randomized. The distribution of observables may have changed differently over time which can introduce bias in the analysis. In addition, the administrative nature of the data introduces limitations. The absence of key clinical details—such as lab values, vital signs, and outpatient prescriptions—limits the accurate assessment of disease severity and pre-hospital diabetes management, which constrains the ability to disentangle potential indirect channels. Although our analysis incorporates patient demographics, insurance type and patient zip code as proxies for socioeconomic and geographic characteristics, these measures may not capture the broader range of social determinants of health, and residual confounding remains possible.

Another limitation relates to the identification of COVID-19 cases. The ICD-10 code that we use to capture COVID-19 comorbidity may have been underreported during the early months of 2020, when coding practices were still being standardized. Although we consider this concern and perform a robustness check that excludes Q1–Q2 2020, some misclassification of COVID-19 status remains possible. Incomplete identification of COVID-19 comorbidity could bias association of outcomes with indirect effects upward.

Our analysis excluding patients with COVID comorbidity has a few weaknesses. First, it assumes that all COVID-19 cases are captured accurately. Even though there were protocols that required anybody entering hospitals to get tested (including healthcare workers) the possibility of false negatives cannot be ruled out. Also, our analysis assumes that the virus affects patients when they are COVID-positive and, therefore, cannot capture the long-run impacts of the disease. Although DKA is more commonly associated with acute COVID-19, evidence suggests that it may contribute to long-term metabolic disturbances, thereby increasing the risk of DKA (48, 49).

Another limitation of our analysis is the inability to capture potential county or city-level policy variations during the pandemic. Local jurisdictions in Texas adopted different preventive measures and public health policies, such as face mask mandates or business restrictions, which may have influenced the spread of the virus, healthcare access, or hospital strain. These local policy shifts could have affected DKA outcomes in ways not fully reflected in our models. Our robustness check, including patient zip code fixed effects, introduces geographic controls that account for time-invariant geographical variations; however, this approach cannot entirely capture county-level policy differences.

Finally, our analysis is based on hospital discharge data from Texas, which may limit our findings’ generalizability. Variations in state-level COVID-19 policies, Medicaid eligibility, insurance coverage, and healthcare infrastructure may lead to differences in the magnitude of direct and indirect effects across settings. For example, Texas implemented relatively limited restrictions and reopened early, which may have reduced healthcare delays compared with states that adopted stricter measures, while simultaneously increasing viral transmission. Texas also has the highest uninsured rate in the nation and has experienced significant rural hospital closures, factors that may amplify access barriers relative to states with broader insurance coverage and denser healthcare networks. On the other hand, studies from other regions document broadly similar patterns of change in DKA frequency and mortality during the pandemic. Although our results should be interpreted with these limitations in mind, they remain consistent with broader evidence on rising DKA incidence and adverse outcomes during the pandemic.

## 6 Conclusion

This study highlights the increased vulnerability among patients with DKA during the pandemic period. While our findings align with existing evidence suggesting a significant association between COVID-19 and elevated DKA mortality, they also point to potential links between the pandemic and mortality through non-viral pathways. Specifically, our results indicate that COVID-19 comorbidity may not fully account for the observed rise in DKA mortality during the pandemic. Notably, DKA patients experienced a significant rise in mortality attributable to factors such as healthcare system disruptions and delays in accessing primary care. Moreover, these indirect effects of the pandemic may have disproportionately affected DKA patients compared to other groups of acute-care patients.

Our findings show the importance of policies that keep primary care and essential medications available for people with diabetes. Expanding telemedicine and making it easier for disadvantaged groups to use these services are important steps, especially during public health emergencies. It is also important to keep medications—especially insulin—affordable and within reach through approaches such as broader insurance coverage, price caps, and emergency refill rules.

## Data Availability

Datasets analyzed in this study are available from the Texas Department of State Health Services: https://www.dshs.texas.gov/thcic/hospitals/Inpatientpudf.shtm. This study uses Inpatient Public Use Data File (PUDF), which contains anonymized and de-identified hospital discharge data from Texas hospitals from 2018 to 2021. While earlier data are freely available, data from 2019 onward require a formal request via the Inpatient PUDF Order Form and may be subject to a fee.
