# Supplementary material for: Pandemic-driven healthcare disruptions and their disproportionate impact on patients with diabetes: evidence from Texas
Source: Front Public Health. 2025 Oct 3;13:1597297. doi: 10.3389/fpubh.2025.1597297 (PMC12531178; doi:10.3389/fpubh.2025.1597297)
Supplement: Supplementary file 1 [file Data_Sheet_1.PDF]

APPENDIX

Figure A1: Sample Selection

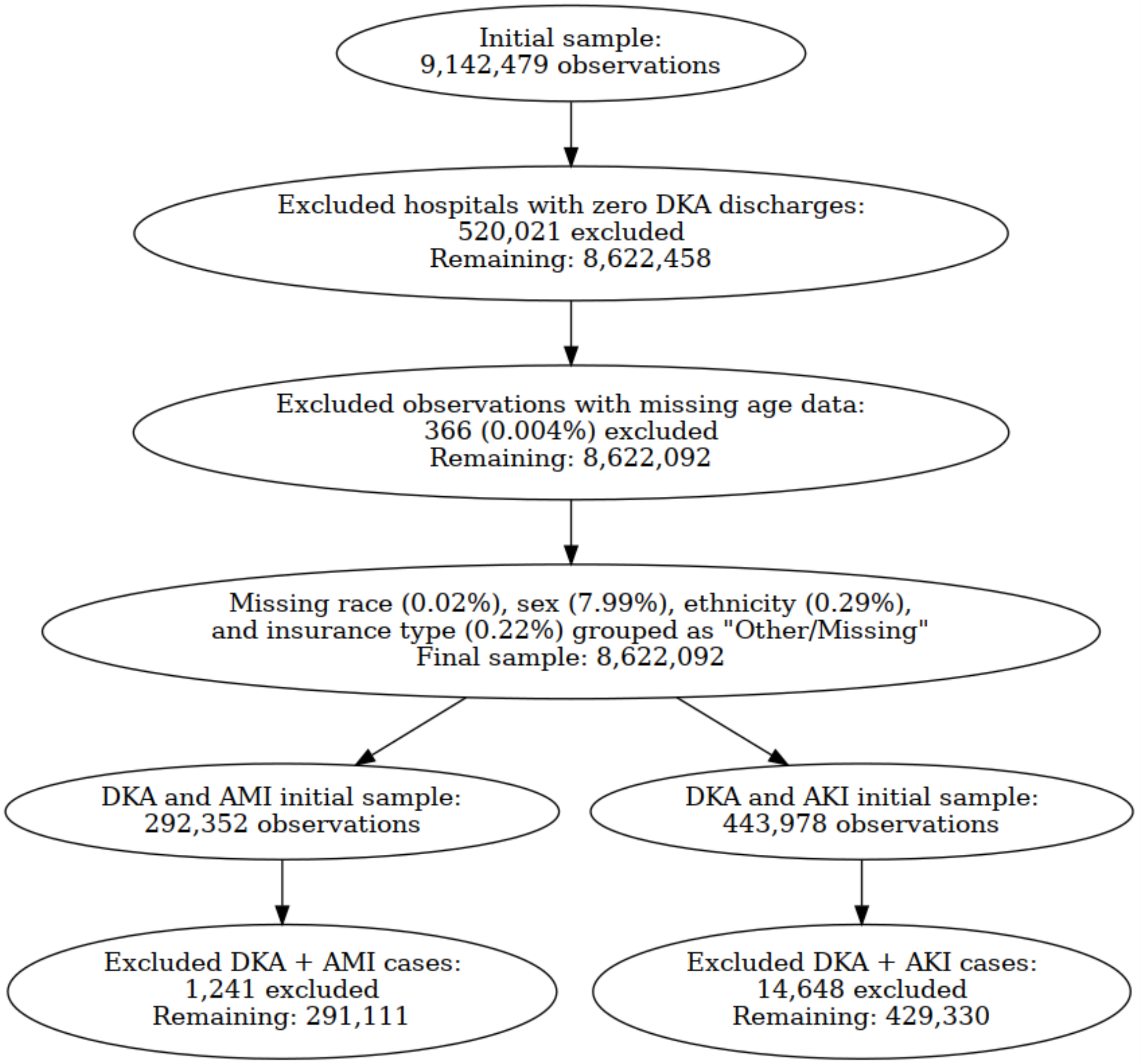

This flow diagram summarizes the sample selection process for the overall dataset and two subgroups (DKA and AMI, DKA and AKI), including exclusions and handling of missing data. Observations with missing information in covariates were dropped if the missing rate was less than 0.01%; otherwise, categorized as 'Other/Missing'.

Table A1: Summary Statistics

	Pre-COVID	Post-COVID	Difference (Post-Pre)
**All Discharges**			
**Age (%)**			
0-17	17.83	16.72	-1.11***
18-44	26.41	26.94	0.52***
45-64	22.99	23.29	0.30***
65-74	15.02	15.33	0.32***
75+	17.75	17.72	-0.03
**Race (%)**			
White	68.55	68.65	0.10***
Black	13.82	14.25	0.44***
Other	17.63	17.09	-0.54***
**Ethnicity (%)**			
Hispanic	29.05	27.05	-2.00***
**Sex (%)**			
Female	53.87	52.89	-0.98***

Male	36.91	37.61	0.71***
Other	9.22	9.50	0.28***
**Insurance Type (%)**			
Medicare	26.40	24.15	-2.24***
Medicaid	19.48	18.97	-0.51***
Private	42.09	44.09	2.00***
Uninsured	10.44	10.69	0.25***
Other	1.59	2.10	0.51***
**Covid (%)**			
All Discharges	0	7.67	
Diabetic Ketoacidosis (DKA) Discharges	0	8.52	
Acute Myocardial Infarction (AMI) Discharges	0	3.61	
Acute Kidney Injury (AKI) Discharges	0	2.58	
**Outcomes**			
DKA per 100,000 population	22.83	25.78	2.95***

**All Discharges**			
Mortality (%)	1.72	2.84	1.11***
Length of Stay (LOS) (days)	4.88	5.22	0.34***
DKA per 1000 discharges	8.95	10.92	1.98***
**DKA Discharges**			
Mortality (%)	0.98	2.54	1.57***
LOS (days)	3.92	4.45	0.53***
**AMI Discharges**			
Mortality (%)	5.39	5.79	0.40***
LOS (days)	5.23	5.18	-0.06**
**AKI Discharges**			
Mortality (%)	1.81	2.47	0.65***
LOS (days)	5.11	5.33	0.21***
**Total discharges**	5,152,332	3,469,760	
**Number of hospitals**	462	459	

Note: *** p<0.01, ** p<0.05. Pre-COVID: 2018q2 – 2019q4. Post-COVID: 2020q1 – 2021q1

Figure A2: Diabetic Ketoacidosis (DKA) Incidence Rate Over Time

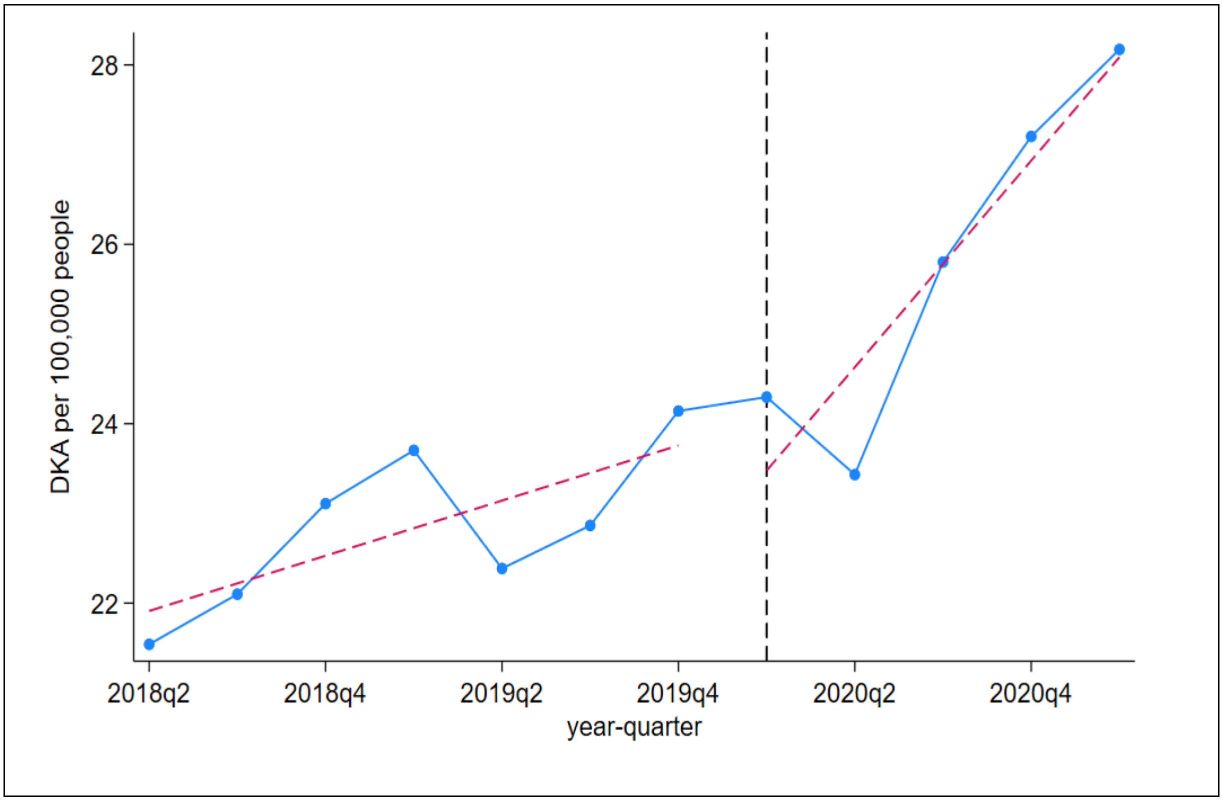

The incidence rate is DKA hospitalizations per 100,000 population.

Figure A3: Diabetic Ketoacidosis (DKA) Frequency and Outcomes Over Time

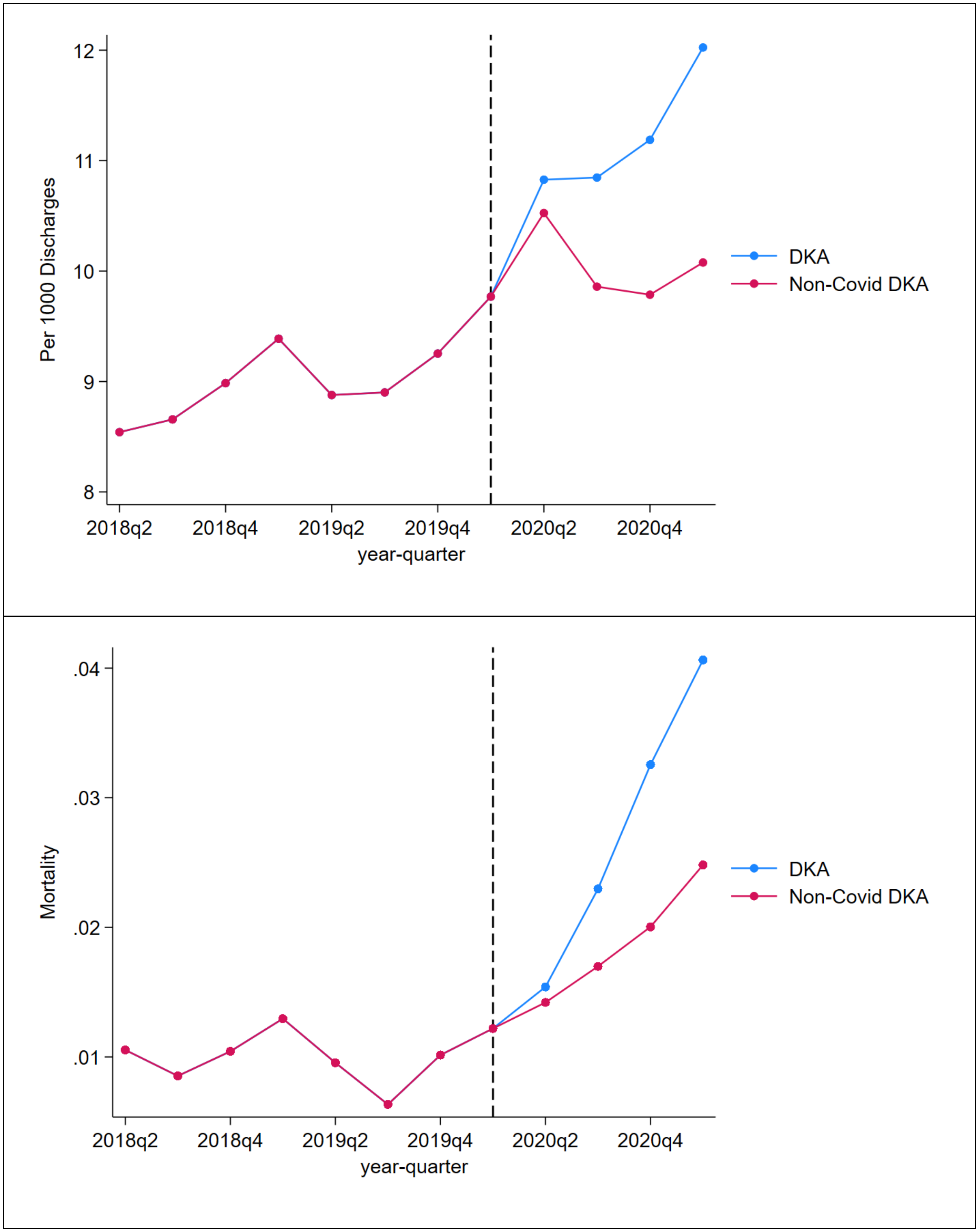

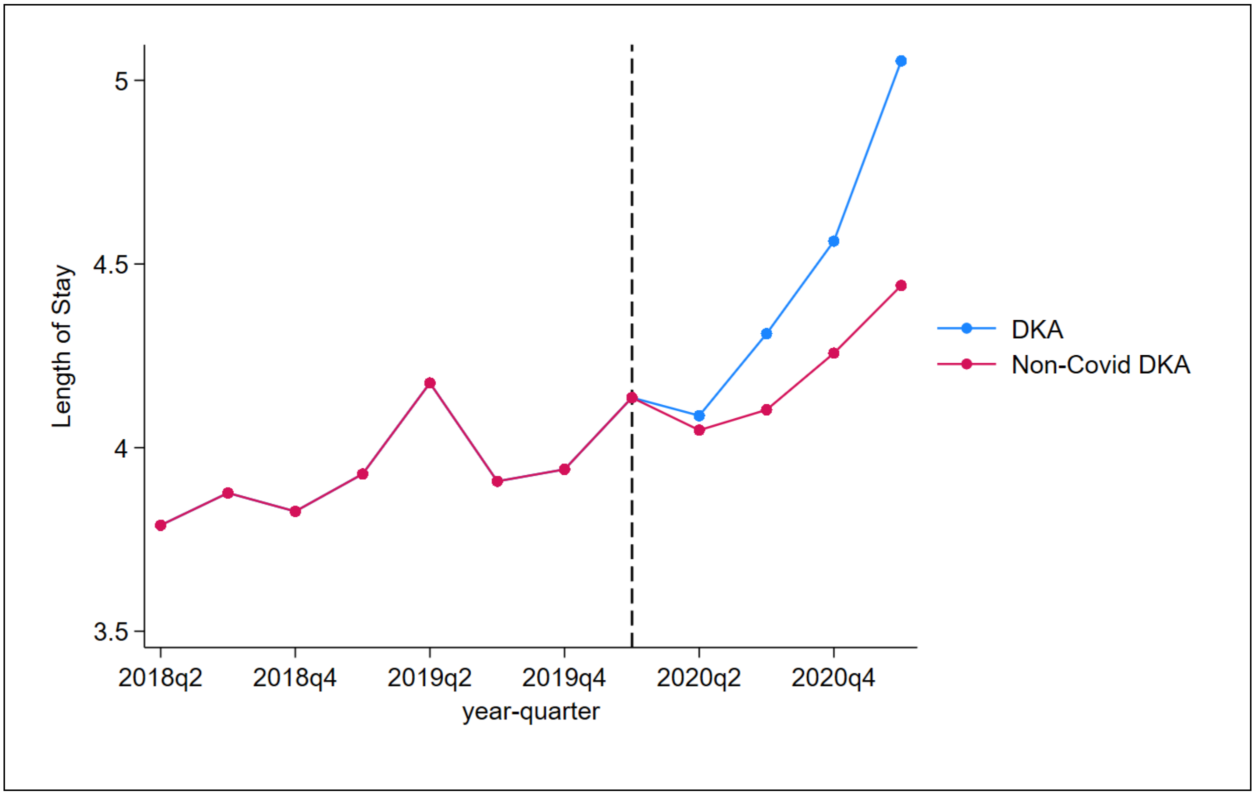

Frequency is DKA incidences per 1000 discharges. Mortality is the average mortality of DKA discharges. Length of stay is the average days of hospital-stays for discharges with DKA. Non-Covid DKA excludes DKA discharges with COVID-19 comorbidity.

Figure A4: Acute Myocardial Infarction (AMI) vs. Diabetic Ketoacidosis (DKA) Mortality and Length of Stay Over Time

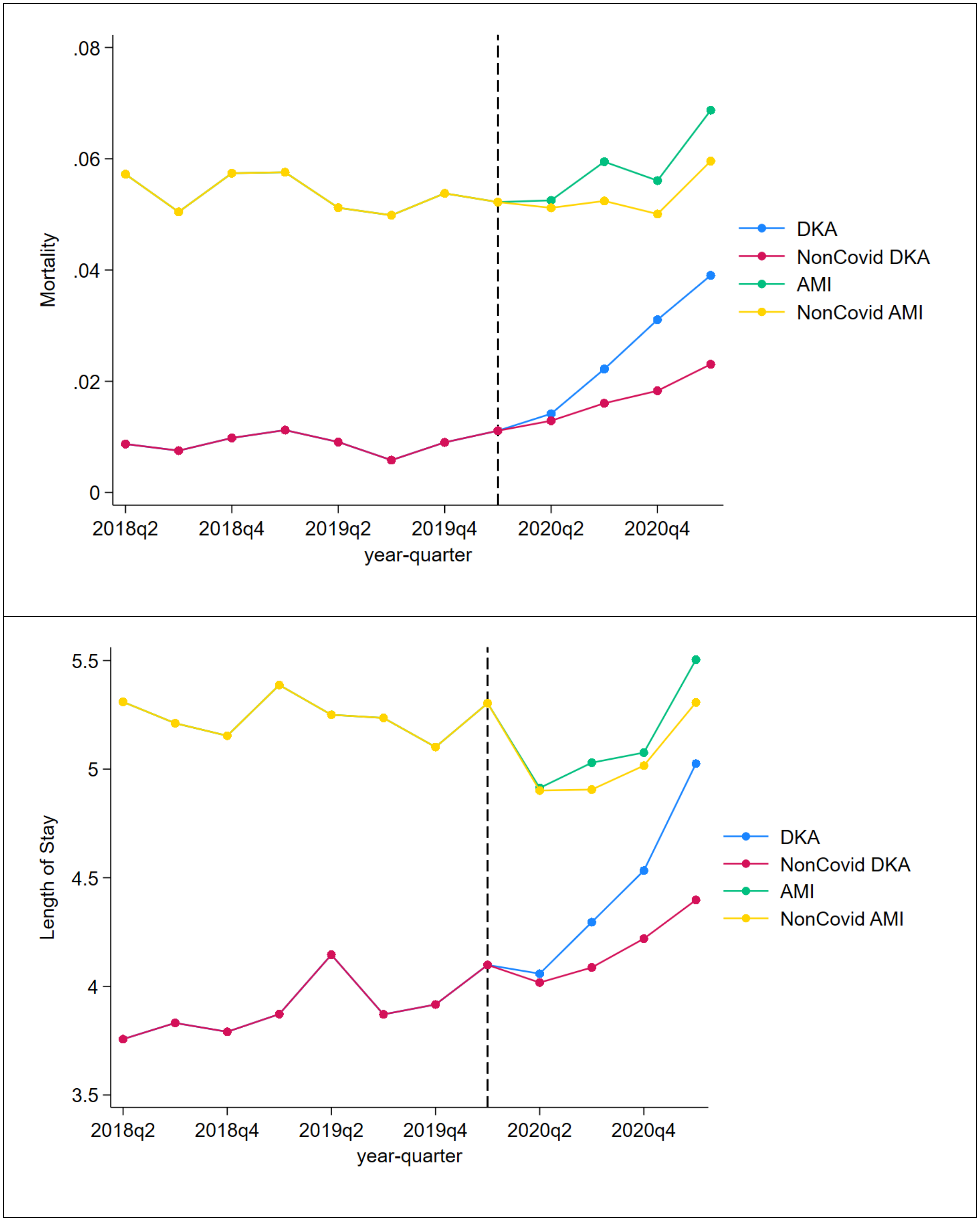

Figure A5: Acute Kidney Injury (AKI) vs. Diabetic Ketoacidosis (DKA) Mortality and Length of Stay Over Time

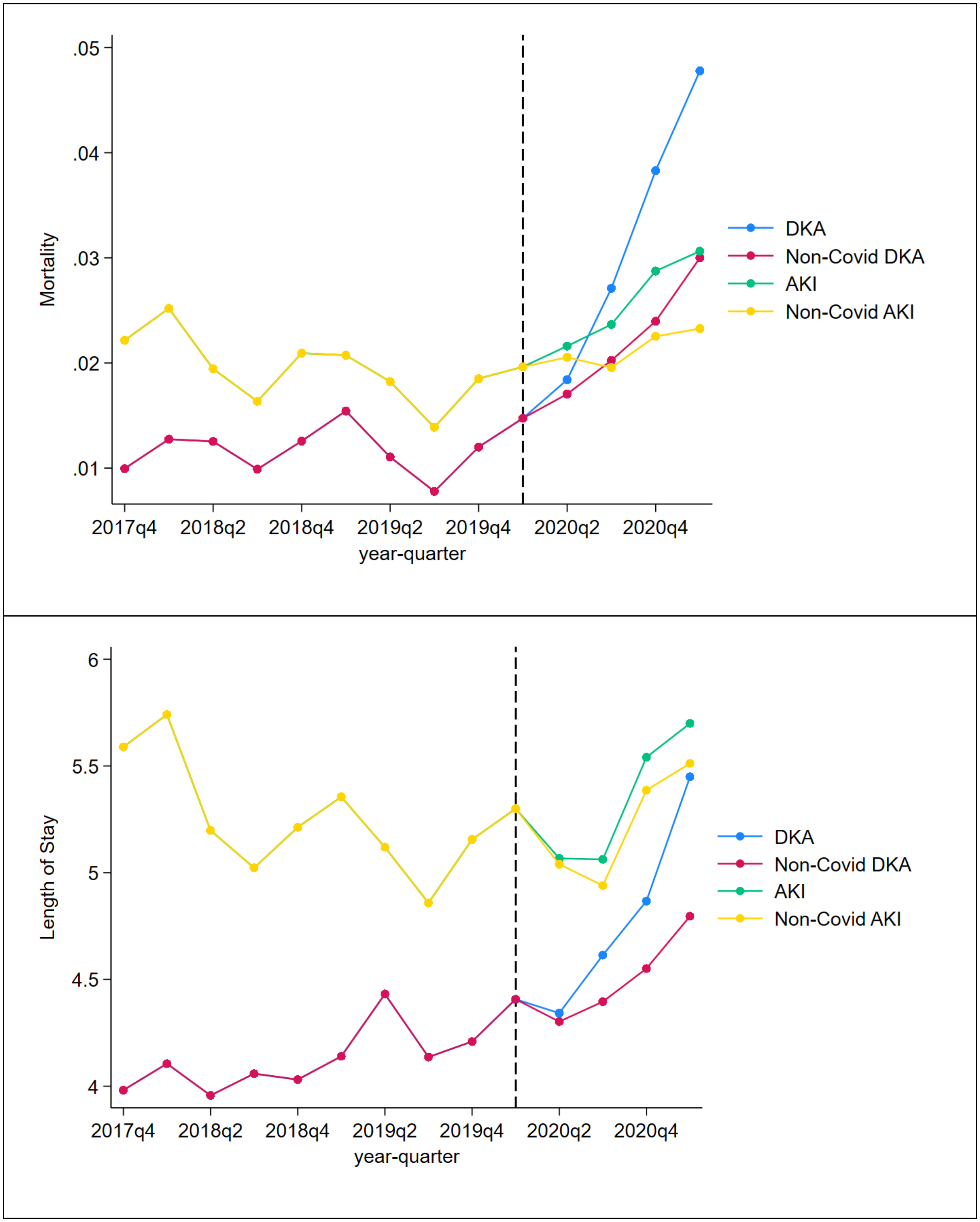

Table A2: Risk of Mortality (%)

	Pre-COVID	Post-COVID	Post-Pre
**DKA**			
0	0	1.34	1.34***
1	49.4	36.31	-13.09***
2	26.16	23.4	-2.75***
3	16.63	24.33	7.7***
4	7.81	14.62	6.81***
**AMI**			
0	0	0.53	0.53***
1	18.06	16.41	-1.65***
2	22.4	21.18	-1.22***
3	33.61	34.08	0.46**
4	25.93	27.8	1.87***
**AKI**			
0	0	0.48	0.48***
1	12.9	5.71	-7.19***
2	33.65	44.19	10.54***
3	43.49	38.83	-4.66***
4	9.96	10.79	0.83***

Risk of mortality score from the All Patient Refined (APR) Diagnosis Related Group (DRG). Indicates the likelihood of dying. 0: Unknown 1: Minor 2: Moderate 3: Major 4: Extreme. Significance of differences (Post-Pre) is tested via two-sided t-tests. *** $p<0.01$, ** $p<0.05$. Pre-COVID: 2018q2 – 2019q4. Post-COVID: 2020q1 – 2021q1
